# Development of a taxonomy to describe massage treatments for musculoskeletal pain

**DOI:** 10.1186/1472-6882-6-24

**Published:** 2006-06-23

**Authors:** Karen J Sherman, Marian W Dixon, Diana Thompson, Daniel C Cherkin

**Affiliations:** 1 Center for Health Studies, Group Health Cooperative, Seattle, Washington 98101, USA; 2 Department of Epidemiology, University of Washington, Seattle, Washington 98195, USA; 3 Private Practice, Portland, Oregon 97214, USA; 4 Private Practice, Seattle, Washington 98112, USA; 5 Departments of Family Medicine and Health Services, University of Washington, Seattle, Washington 98195, USA

## Abstract

**Background:**

One of the challenges in conducting research in the field of massage and bodywork is the lack of consistent terminology for describing the treatments given by massage therapists. The objective of this study was to develop a taxonomy to describe what massage therapists actually do when giving a massage to patients with musculoskeletal pain.

**Methods:**

After conducting a review of the massage treatment literature for musculoskeletal pain, a list of candidate techniques was generated for possible inclusion in the taxonomy. This list was modified after discussions with a senior massage therapist educator and seven experienced massage therapists participating in a study of massage for neck pain.

**Results:**

The taxonomy was conceptualized as a three level classification system, principal goals of treatment, styles, and techniques. Four categories described the principal goal of treatment (i.e., relaxation massage, clinical massage, movement re-education and energy work). Each principal goal of treatment could be met using a number of different styles, with each style consisting of a number of specific techniques. A total of 36 distinct techniques were identified and described, many of which could be included in multiple styles.

**Conclusion:**

A new classification system is presented whereby practitioners using different styles of massage can describe the techniques they employ using consistent terminology. This system could help facilitate standardized reporting of massage interventions.

## Background

Massage therapy, the manual manipulation of soft body tissues to enhance health and well-being, is one of the oldest forms of medicine known to mankind and has been practiced worldwide since ancient times [1]. Today, more than 80 different forms of massage have been identified, many developed in the last 30 years. Although massage is used for a variety of specific reasons (e.g., relaxation, comfort at the end of life, relieving pain, enhancing athletic performance), it is undertaken with the general goal of helping the body achieve or increase health and well-being. Touch given with the intention of healing, the common factor in all forms of massage, is believed not only to have beneficial effects on tissue, body fluids and other systems of the body but also to communicate caring with another human being [2,3]. Massage therapists believe the healing power of touch, common to all of the forms of massage, is a fundamental factor in their success in facilitating their clients' goals for health.

With the burgeoning interest in complementary and alternative medicine (CAM) in developed countries has come increased scientific interest in undertaking studies of CAM therapies, including massage. Such studies have revealed a lack of fundamental information on many CAM therapies that has impeded the research process. One of the challenges in conducting research in the field of massage and bodywork is the lack of consistent terminology for describing the treatments practitioners employ. This absence of a common language makes it difficult to ensure that different massage therapists are consistently describing what they are actually doing in treatment sessions and that research protocols for massage studies are reproducible. This polyglot results, at least in part, from the numerous trademarked styles of massage that have been developed and taught by individuals who use specialized language to describe their component techniques (i.e., specific physical manipulations of tissue) and from using techniques developed by other disciplines. Some massage styles with different names may be essentially the same (e.g., Structural Integration and Rolfing^®^). However, some commonly used styles of massage therapy, including "deep tissue" and neuromuscular therapy, are not consistently defined. For example, some practitioners consider "deep tissue" work to be a synonym for neuromuscular therapy, while others consider "deep tissue" to mean the application of Swedish massage strokes with strong pressure, the application of acupressure, or the use of myofascial release [4]. Finally, the same technique or stroke is often given different names in different styles (e.g., deep effleurage, muscle sculpting, and longitudinal friction are the same), so massage therapists with different training may not realize when they are applying the same technique.

As part of a study of massage for neck pain, we surveyed the massage treatment literature and developed a taxonomy of techniques using neutral language to describe what is being done to the body. In this report, we describe the development of our taxonomy, including the appropriate application of "intent" or purpose, and illustrate its use in a study evaluating massage for neck pain. Finally, we discuss the strengths and weaknesses of our taxonomy and provide suggestions for its future refinement.

## Methods

One of the authors (MWD), an experienced massage educator, reviewed 18 textbooks [1,3,5-20] and 3 databases (Massage Therapy Foundation database, Bodywork Knowledgebase and MEDLINE) that include descriptions of the commonly used styles of massage and their component techniques used to treat patients with musculoskeletal pain. Using those sources and her general knowledge of the field, she generated a list of candidate techniques. Each candidate technique included a mechanical description of the stroke using neutral descriptive language, notes regarding appropriate application (e.g., the anatomic locations where it could be applied, types of conditions) and examples of the massage styles that might use this technique. She produced a document that was then shared with another author (DT) and they discussed and resolved any differences. The document was further refined during discussions with the seven massage therapists who were treating patients in the neck pain study. A subset of the techniques was then incorporated into the protocol for the neck pain study.

## Results

The proposed taxonomy was conceptualized as a system with three levels of detail: principal goals of treatment, styles and techniques (Table 1). At the most general level, four principal goals of treatment can be elucidated: 1) to promote relaxation and wellness (relaxation massage), 2) to address clinical concerns (clinical massage), 3) to enhance posture, movement and body awareness (movement re-education), and 4) to balance and "move" subtle energy (energy work). Each of these goals can be accomplished using a number of different styles of massage, some of which are trademarked (e.g., Rolfing^®^). In a typical massage therapy session, more than one goal is addressed and when addressing the broad goals described above, more than one style of massage is used in a course of treatments and even within a single treatment session. Finally, a single style of massage may be used to address different goals. For example, Structural Integration, can be used to enhance athletic performance (relaxation massage), address a clinical condition such as scoliosis (clinical massage) or improve posture (movement re-education).

**Table 1:** Proposed taxonomy of massage practice

Level				
Principal Goals of Treatment	Relaxation Massage	Clinical Massage	Movement Re-education	Energy Work
Intention	Relax muscles, move body fluids, promote wellness	Accomplish specific goals such as releasing muscle spasms	Induce sense of freedom, ease and lightness in body	Hypothesized to free energy blockages
Commonly Used Styles (examples*)	Swedish massage, spa massage Sports massage	Myofascial trigger point therapy Myofascial release Strain counterstrain	Proprioceptive Neuromuscular Facilitation Strain counterstrain Trager	Acupressure Reiki Polarity Therapeutic Touch Tuina
Commonly Techniques (examples**)	Gliding Kneading Friction Holding Percussion Vibration	Direct pressure Skin rolling Resistive stretching Stretching – manual Cross-fiber friction	Contract-relax Passive stretching Resistive stretching Rocking	Direction of energy Smoothing Direct pressure Holding Rocking Traction

* While some styles of massage are commonly used in addressing one of the four principal treatment goals, some may be used to address several distinct treatment goals.

** By varying the intent (or purpose) for a technique, many of them can be used in massages with different principal treatment goals.

Typically, massage students are taught a sample of styles in their basic massage training and then enhance their skills and learn other styles through continuing education workshops. Styles, consisting of a number of distinct techniques, are commonly distinguished by a unique combination of these techniques, and by the underlying intent of when and why to apply the techniques. Specific techniques, which refer to physical manipulations of the tissue, are the most fundamental and specific level of classification and are the building blocks of each of the styles (Additional file 1). Specific techniques are commonly used in more than one style of massage and may be practiced by other professionals, such as osteopaths, physiotherapists, and movement educators. Principal goals, styles and techniques are discussed in more detail below.

### Principal goals of treatment

*Relaxation massage *is massage that is specifically given to relax the body and promote wellness. Relaxation massage has the intention of moving body fluids (such as lymph and blood), nourishing cells, removing wastes from cells, relaxing muscles and diminishing any pain. In the US, the most widely taught and practiced style of relaxation massage is Swedish massage [1,3], which employs five basic strokes: effleurage (gliding), petrissage (kneading and lifting), friction (moving the tissue layers underneath the skin), vibration, and percussion. Other common styles include spa massage and sports massage. Relaxation massage may include styles of massage that are more commonly used to address non-relaxation goals if such styles are applied with the intent to relax the body. For example, lymphatic drainage, commonly used as part of clinical massage (e.g., to reduce inflammation), is believed to be effective in stimulating the parasympathetic nervous system to promote relaxation.

*Clinical massage *involves more focused manipulation of the muscle and/or surrounding fascia and may address other systems in the body such as lymphatic, circulatory and nervous systems [3]. Its intent is to relieve pain and restricted movement. Popular styles of clinical massage are myofascial trigger point therapy, myofascial release, neuromuscular therapy and Structural Integration or Rolfing^®^. They differ from relaxation massage because they include focused therapeutic goals (e.g., releasing muscle spasms, strengthening or stretching specific muscles and remodeling fascia). Clinical massage may include styles of massage often used for other principal goals. For example, Muscle Energy Technique, often used for enhancing ease of movement (movement re-education), can also be used as a clinical technique, for example, to reduce muscle spasms in a patient with whiplash.

*Movement re-education *emphasizes using movement to enhance posture, body awareness and movement [3]. Movement re-education is generally intended to induce a sense of freedom, ease and lightness in the body. Some styles of movement re-education focus on active exercises to teach healthier ways of moving (e.g, Alexander technique, Trager^®^, Feldenkrais^®^). These styles may be used by non – massage therapists. Other styles focus on tablework in which the practitioner induces, assists or resists movement for a patient (e.g., Proprioceptive Neuromuscular Facilitation, Muscle Energy Technique, strain counterstrain). Some styles of massage commonly used for a different treatment goal, can be used to increase function and movement (e.g., sports massage).

*Energy work *(also called subtle energy techniques or body-mind therapies) are believed to "assist the flow of energy in the body" by employing very light touch or by holding the hands just above the skin [6]. These include Reiki, Polarity and Therapeutic Touch as well as massage traditions deriving from Eastern cultures, such as acupressure, Amma, Shiatsu and Tuina. The intention of energy work is to move stagnant or blocked "energy" so it can circulate freely throughout the body.

### Styles

The previous section presented examples of the more common styles of massage used to address each of the principal goals of massage. In this section, we present more detailed information on two common styles of massage that are often used when addressing clinical concerns: myofascial release and neuromuscular therapy.

Myofascial release is a style of manual therapy that Barnes [6] defines as a "whole body, hands-on approach for the evaluation and treatment of the human structure. Its focus is to optimize the function of the fascial system." Component techniques of myofascial release include three techniques used in craniosacral therapy (compression – static, listening to and following the craniosacral rhythm, still point), in addition to cross-fiber friction, deep gliding, holding, J-stroke, manual stretching, traction, skin rolling, rocking, jostling, shaking and vibration [6,14] (see Additional file 1).

Neuromuscular therapy focuses on relieving local dysfunctions of the tissue, including trigger points, ischemia, inflammation, muscle hypertonia, and nerve impingement. It includes 15 component techniques: application of cold and heat, compression, cross-fiber friction, direct pressure, friction, J-stroke, manual stretching, percussion with stretch, scraping, stripping, and vibration focused on trigger points, and three types of resistive stretching: lengthening, contracting the agonist; lengthening the agonist, contracting the antagonist; and lengthening the agonist, contracting agonist and antagonist. [14,19] (see Additional file 1).

### Techniques

Our literature review identified 36 techniques, where techniques are considered to be the "discrete" and "distinct" strokes and manipulations performed by a massage therapist (e.g., static friction, deep gliding, skin rolling) (Additional file 1). Techniques are the "building blocks" of a massage treatment session. Techniques are listed in alphabetical order and include descriptions as well as examples of styles wherein these techniques have been commonly used. In some cases, techniques may be related, either because they are similar in how they are performed (e.g., rocking, jostling, shaking, vibration) or because they are components of a single style of massage (e.g., circular compression and rebound are techniques used in lymph drainage). In those situations, the related techniques are listed together (and designated by one or more capitalized letters before the name of the technique). Three of these techniques involve application of a foreign substance on or near the body (e.g., heat or cold) and one technique, directed breathing, could be performed by the patient as part of many other techniques.

Techniques may be used with different intentions that depend on the circumstances in which they are performed. For example, the technique of direct pressure may be used in trigger point therapy to release trigger points, in sports massage to decrease pain and muscle spasms and in neuromuscular therapy to soften adhesions and make them more pliable. Also, the technique of holding could be used to relax the tissues in Swedish massage, to warm tissues before stretching them in myofascial release, and is hypothesized to "balance energy" in polarity therapy. Finally, the technique of positional release is used in sports massage to restore normal muscle length and in Aston patterning to convey a sense of ease and comfort and body awareness.

#### Application of classification system to neck pain study

In this section, we illustrate the use of our proposed classification system in our neck pain study. The protocol for our neck pain study was designed to allow massage therapists sufficient flexibility in their application of techniques to provide appropriate care for each patient's presenting condition, while remaining within the realm of "common practice". Massage therapists were permitted to use up to 10 60-minute treatments for each patient over a 10 week period.

Even though the study goal was to evaluate massage as a treatment for neck pain, which clearly falls within the principal goal of clinical massage, our protocol (Table 2) was quite broad. However, we excluded styles of massage originating in Asia because most are not commonly taught to massage therapists and because Shiatsu has been associated with serious adverse events in patients with neck pain [21]. Consistent with our focus on clinical massage, we also limited the use of energy work (e.g., Reiki) to less than 20% of each treatment session. Finally, we restricted the use of some movement re-education techniques (i.e., those that use active movement) and excluded a subset of clinical massage that involved formulaic styles such as Rolfing or Hellerwork, although we permitted use of individual techniques that are used in these styles.

**Table 2:** Components of treatment protocol in study of massage for neck pain

*1. Styles or techniques allowed with no restrictions*
1. Application of cold
2. Application of heat
3. Compression – pumping or static
4. Craniosacral
5. Friction or direct pressure
6. Cross-fiber friction
7. Gliding (effluerage) – Swedish
8. Gliding – deep (effluerage, stripping) – clinical
9. Holding
10. Kneading (petrissage)
11. Lymphatic drainage
12. Percussion (tapotement)
13. Rocking, jostling, shaking, vibration
14. ROM – active or resistive (also active assisted and/or resisted stretching, MET, PNF – consisting of three types of resistive stretching: lengthening, contracting the agonist; lengthening the agonist, contracting the antagonist; and lengthening the agonist, contracting agonist and antagonist)
15. ROM – passive (passive stretching, positional release)
16. Skin rolling
17. Stretching (manual)
18. Traction
19. Trigger point therapy

*2. Principal treatment goals LIMITED by protocol *(*i.e., allowed only if represent less than 20% of an individual session*)
**Energy Work **(e.g., Reiki, Polarity, Therapeutic touch, Zero Balancing)
**Movement Education (Active Exercise Styles) **(e.g., Alexander Technique, Feldenkrais Method, Aston Patterning)

*3. Treatments proscribed by protocol*
Aromatherapy
Asian bodywork (shiatsu or other meridian based massage)
Dietary supplements
Recipe techniques – although components of those may be acceptable

We were motivated to collect information on what the massage therapists actually did during the session, by the desire to describe their treatment in as simple and unambiguous terminology as possible and at the level of detail necessary to reproduce the treatments. Therefore, we did not always ask the therapists to record their treatment details using one of the 36 techniques shown in Additional file 1. Our protocol allowed therapists using craniosacral therapy and manual lymph drainage (two styles which are not focused on the treatment of muscle or general connective tissue) in a session to record these as styles because these two styles typically use their full set of techniques in a massage session. In addition, we included three distinct sets of "related techniques" (i.e., 1) rocking, jostling, shaking, vibration; 2) active or resisted range of motion, which consists of three types of resistive stretching: i) lengthening, contracting the agonist, ii) lengthening the agonist, contracting the antagonist, and iii) lengthening the agonist, contracting agonist and antagonist, and 3) passive range of motion, which consists of passive stretching and positional release) due to the similarity in application and anticipated results of the "related techniques". The 13 specific techniques were: application of cold; application of heat; compression; cross-fiber friction; friction or direct pressure; gliding (effluerage) – Swedish; gliding – deep (deep effluerage, longitudinal friction, stripping) – clinical massage; holding; kneading (petrissage); percussion (tapotement); skin rolling; stretching – manual; traction. Finally, the last element of the protocol, trigger point therapy, was a subcategory of friction or direct pressure technique with a specific intention (i.e., applied to trigger points). We chose to distinguish the application of direct pressure to trigger points from the application of direct pressure to other soft tissue components because relief of trigger points was anticipated to be an important part of the massage treatment for neck pain. Thus, our protocol involved substantial detail on the techniques most likely to be applied to muscles and general connective tissue. While our protocol also permitted self-care recommendations, a discussion of those is beyond the scope of this manuscript.

## Discussion

This taxonomy is a first step in enabling researchers and massage therapists to more clearly communicate about the nature of the massage treatments they are giving by using common language describing specific techniques that may have originally been learned as part of training in different styles of massage. Even though only two massage therapists were involved in the development of this taxonomy, seven others were involved in a study that employed many of the described techniques and were comfortable using them to characterize the component techniques of their sessions. We believe, however, that future development of this taxonomy should include feedback from a broader range of massage therapists in the US and other Western countries as well as from manual medicine practitioners, such as osteopaths and physical therapists, who have different basic training.

We expect that our taxonomy will offer practical applications for communication among massage therapists in their educational process. Students receiving their basic training in massage school would benefit from having the entire faculty use the same language to describe a particular technique and using neutral language describing hand movements could help students more clearly understand what they are doing. Consistent terminology would make it easier for US students who are taking the national certification exam in massage administered by the National Certification Board for Therapeutic Massage and Bodywork, which is required for licensure in at least 24 states [22]. We also believe that participants in continuing education programs, which are required for continued licensure in many states, would benefit from the consistent terminology.

Future clinical research studies, regardless of whether they are evaluating massage as a whole system of care or investigating specific styles of massage, may benefit from creating a video or DVD that illustrates the application of specific techniques in a variety of circumstances to ensure that there is no confusion among massage therapists about each hands-on technique. Alternatively, researchers could provide training sessions to illustrate the specific techniques to ensure that terminology was being used consistently. Because our taxonomy was developed in the context of a study for neck pain, it will likely be most useful when describing the treatment of musculoskeletal conditions. While these are among the most common reasons people seek out massage [22], we believe that additional refinement of this taxonomy would be needed to extend its use in studies of such topics as massage for cancer patients, geriatric patients or for somatic psychotherapy.

Although various descriptions of massage exist [3, 15], we are unaware of other taxonomies that use a multi-level system of classification, include the concept of "intent", and that describe what massage therapists are actually doing in a way that is reproducible. Our example of the use of this taxonomy for the development of treatment protocols in our neck pain study illustrates how researchers need to carefully consider the condition being treated and the likely nature of the treatment in order to specify the protocol in sufficient detail. However, developing treatment protocols for massage studies involves more than simply specifying the styles and/or techniques to be included in the study. Researchers will need to consider the qualifications of the massage therapists providing treatment and specify any restrictions regarding: 1) the anatomic sites to be massaged, 2) the pressure applied using various techniques, and 3) the intention to be used with specific techniques.

Finally, we wish to emphasize that massage therapists recognize the importance of creating a warm and caring relationship in the healing process and that they regard touch itself as a central healing feature of all techniques they employ. One consequence of their training is that massage therapists do not believe that a "placebo" massage exists and thus researchers who wish to study massage within its own worldview would need to design studies with more appropriate controls than, for example, light touching. Even though many challenging issues regarding design and conduct of studies of massage therapy remain, our proposed taxonomy provides an initial step toward standardized descriptions of massage interventions. We suspect that such standardization will be useful for studying massage from a wide variety of perspectives.

## Conclusion

Massage therapists have typically used inconsistent terminology to describe their treatments. We propose a classification system whereby practitioners using different styles of massage can describe their techniques using common language. This system could serve as an important step in facilitating standardized reporting of massage interventions for descriptive and clinical studies.

## Competing interests

The author(s) declare that they have no competing interests.

## Authors' contributions

KJS obtained funding, participated in the conception of the taxonomy and drafted the manuscript; MWD conceived of creating the taxonomy, reviewed the literature and drafted the taxonomy, and participated in discussions of the manuscript content; DT reviewed the taxonomy, lead the discussion of the neck pain protocol with the massage therapists in the neck pain study and participated in discussions of the manuscript contents; DC participated in the design of the overall study and revised the manuscript for critical intellectual content. All authors read and approved the manuscript.

## Pre-publication history

The pre-publication history for this paper can be accessed here:



## Supplementary Material

Additional File 1 Massage therapy techniques. A list of the 36 distinct techniques that form the third level of our taxonomy. Click here for file
