# Supplementary material for: Development of a taxonomy to describe massage treatments for musculoskeletal pain
Source: BMC Complement Altern Med. 2006 Jun 23;6:24. doi: 10.1186/1472-6882-6-24 (PMC1544351; doi:10.1186/1472-6882-6-24)
Supplement: Additional File 1 — Massage therapy techniques. A list of the 36 distinct techniques that form the third level of our taxonomy. [file 1472-6882-6-24-S1.doc]

Additional File: Massage therapy techniques

| **Technique*** | **Mechanics of stroke** | **Application notes (e.g, anatomic locations, conditions, pressure)** | **Example styles where used** |
| --- | --- | --- | --- |
| Application of cold | Local application of cold/ice (e.g. compress, ice pack) over protected body part, often for 15 minutes or less. Includes ice massage. | Used over areas of acute inflammation, pain or stiffness. Generally not used over areas of chronic inflammation. | Spa Massage; Sports Massage |
| Application of heat | Local application of heat (e.g. compress, poultice) over protected body part, often for 5 minutes or less. | Used for chronic inflammation. Not used over areas of acute inflammation. | Spa Massage; Sports Massage |
| Application of cold and heat (Vascular flush) | Alternating application of cold (e.g., 3 minutes) with application of heat (e.g., 1 minute). | Used for subacute inflammation. | Neuromuscular Therapy; Sports Massage; Spa Massage |
| APR-ROM –Resistive stretching: lengthening, contracting the agonist (includes contract – relax and “Post Isometric Relaxation”**) | Lengthen muscle until the first resistance barrier, then coach patient to voluntarily resist AGAINST the lengthening for 5-15 seconds. Allow the patient to relax for 5-15 seconds and then either repeat the resistance against the lengthening at the first barrier or lengthen the muscle until you reach the next resistance barrier and repeat the process. This process can be repeated a total of 2-4 times. You want to end with a stretch at the end range of motion. | Recommended for subacute and chronic conditions. May be used for acute conditions with mild resistance. | Muscle Energy Technique (MET);  Proprioceptive Neuromuscular Facilitation (PNF) |
| APR-ROM –Resistive stretching: lengthening the agonist, contracting the antagonist (“Reciprocal Inhibition”**) | Lengthen muscle until the first resistance barrier, then coach patient to voluntarily lengthen the muscle against resistance for 5-15 seconds. As patient relaxes, take the muscle into a greater lengthening and repeat the process 2-4 times. End with a stretch at the end range of motion. | Used in acute conditions. If contracting the agonist is painful, use this technique because the antagonist is the one doing the contractions. | MET; PNF |
| **APR-ROM -**  Resistive stretching: lengthening the agonist, contracting agonist and antagonist (Contract Relax Antagonist Relax) | A third type of resistive stretching that alternates contracting the agonist and contracting the antagonist through a series of barriers. End with a stretch at the end range of motion. | Recommended for subacute and chronic conditions. May be used for acute conditions with mild resistance | MET; PNF |
| Compression **-** pumping | Usually pumping the muscle (and surrounding fascial layer) against the bone – rhythm and force vary with the resistance of the soft tissues. Can also use light pumping to contact and move fluid through the lymph vessels toward the heart. | Generally used over muscles and bone. Avoid the face and kidney. Can also be used for fluid movement with a light touch. | Joint Mobilization; Myofascial Release;  Tuina |
| Compression – Static (origin – insertion technique; sutural releases;  unwinding) | Mechanically shortening a muscle or fascia or joint by pressing on it (origin – insertion technique). Can involve following the body’s natural movement (e.g., unwinding, direction of ease) | Everywhere. In craniosacral therapy, involves very light traction or compression (approximately a dime’s worth or less of pressure) applied in a specific direction determined by the suture between the relevant cranial bones. | Craniosacral;  Myofascial Release; Sports Massage |
| CS - Listening and following the craniosacral rhythm | Hands meld with the client's cranium (or sacrum) or other body area and massage therapist feels the movement of their hands. | Everywhere. | Craniosacral |
| CS - Still point | Monitor the craniosacral rhythm at the client’s cranium or sacrum or other body area and the movement of the craniosacral rhythm is resisted by compression or traction of the therapist’ hands (approximately a dime’s worth or less of pressure). | Everywhere. | Craniosacral |
| Directed breathing | Instructions on the length of inhalation and exhalation, often including instruction to relax while exhaling | Used in conjunction with manual or manipulative techniques. | Acupressure; MET; Shiatsu; Swedish |
| E - Direction of energy (e.g., holding with hands on or off the body; V-spread) | Send positive, healing, balancing intent to a targeted area. May either be focused on a specific physiological intent or on nondirected good will. Many massage therapists send positive intent whenever they perform a massage, even when using clinically oriented techniques. | Everywhere . | Craniosacral; Reiki; Polarity |
| E - Smoothing | Pass the hands over the patient's body (without physical contact) moving toward their feet or towards their head. Imagine the person with an "aura" and try to smoothe the energy of the aura so it is the same density and thickness throughout (can use a tool, e.g., a feather). | Everywhere. | Reiki; Polarity; Therapeutic Touch |
| F – Cross-fiber Friction (transverse friction) | A type of friction that involves moving the patient's skin over the underlying tissue perpendicular to the muscle fibers. | Used in localized areas only, most commonly musculotendinous junctions, tinoperiosteal junction, muscle belly and tendons. Not appropriate for face, use caution if over acute inflammation or tender areas. | Neuromuscular Therapy; Cyriax Friction |
| F - Direct pressure/  static friction | Presses or leans deeply into an area without moving the hands | Nearly everywhere, including trigger points, except the face and areas of acute inflammation. Use caution on tender areas. | Acupressure; Neuromuscular Therapy; Shiatsu;  Swedish |
| F - Friction | Pressing into the skin and moving it over the underlying tissues. Fingers do not glide (as in effleurage) or grasp (as in petrissage). Motions can be circular (ellipsoidal) or in the same direction as the muscle fibers (longitudinal). | EverywhereUse caution on the face and tender areas. | Neuromuscular Therapy; Swedish; Tuina |
| F - Gliding – Deep (deep effleurage, longitudinal friction, stripping) | A type of friction that involves pressing into the skin and moving it over the underlying tissues. Motion follows the direction of the muscle fibers. | Everywhere. Use caution on the face. | Myofascial Release; Sports Massage; |
| F - Scraping | A type of friction that involves scraping bony or ligamentous areas with thumb, knuckles, fingers, or massage tool - smoothing the surface, as if shaving ice. | Joints, bony or ligamentous areas. Avoid the face. . Use caution in tender areas. | Neuromuscular Therapy; Structural Integration |
| F - J-stroke | A type of friction that involves pressing into the skin to underlying tissue and hook that tissue in a J motion. | Use in localized areas, but everywhere on the body, except the face. Use caution on tender areas. | Neuromuscular Therapy; Mysofascial Release;  Structural Integration |
| Gliding (effleurage) | Fr "to skim", to touch lightly on" - palm, knuckles, fingers, or backs of hands glide gradually over client's body. Can be used to move blood and lymph | Everywhere. If moving lymph, long light gliding strokes should be performed in the direction of the heart | Swedish; Lymphatic Drainage |
| Hand rolling | Rotating wrist on body with knuckles as contact point. | Everywhere, except the face. | Lomilomi; Sports Massage; Tuina |
| Holding | Static, broad contact (e.g., whole hand) to warm, relax or mobilize tissues. | Everywhere. | Myofascial Release; Polarity; Reiki; Swedish |
| Kneading (petrissage) | Fr "to knead" - rhythmic lifting , kneading and squeezing of the soft tissue. Can also be used to “milk” lymph fluid and move it toward the heart. | Everywhere. When working on limbs begin proximally. When moving lymph fluid, apply kneading with a light touch. | Sports Massage; Swedish; Tuina |
| K - Skin rolling | A specific type of petrissage where the superficial fascia is grasped between thumb and forefingers (or between other parts of the therapist's body), continuously lifted and rolled over the underlying tissue in a wave-like motion. | Everywhere there are subcutaneous fascial adhesions. | Myofascial Release; Sports Massage |
| LD -Compression - circular | Light to deep circular movements over lymph nodes. | At watershed areas (e.g., axilla, inguinal). | Lymphatic Drainage |
| LD - Pumping | Contacting and moving fluid through the lymph vessels (back to the heart) using repetitions of a pumping or kneading movement. | Along lymph vessels. | Lymphatic Drainage |
| LD - Rebound | Patient inhales slowly and the therapist gradually increases pressure to target area (e.g. lymph nodes in viscera). During patient’s exhale, the pressure is rapidly released. | Abdomen, primarily. | Lymphatic Drainage; Visceral Massage |
| Percussion (Tapotement) | Series of brisk blows, rapidly following each other. | Everywhere, except the throat, breasts, abdomen, bony prominences, kidneys, back of knee, spine or any area of endangerment. Use caution on the face (e.g. light tapping only) | Sports Massage;  Swedish; |
| Percussion with stretch | A series of brisk blows, rapidly following each other applied while muscle is placed/held in the lengthened position. | Used on trigger points and muscle bellies. Use with caution on tender areas. | Sports Massage;  Neuromuscular Therapy |
| P-ROM -Passive stretching | Taking patient's muscle into a position of lengthening by moving a joint without any effort on their part. | Arms, legs, trunk and neck. | Sports Massage; Swedish gymnastics |
| P-ROM -Positional release | Passively placing the body in a position of maximal comfort. Can use point tenderness as a guide to positioning. | Everywhere. | Aston patterning; Craniosacral;Strain counterstrain |
| Stretching –manual – direct | Mechanically lengthening the muscle and/or fascia between your hands without moving a joint. Does not need to be applied parallel to fiber direction. | Everywhere, except not appropriate for face. | Sports Massage; Neuromuscular Therapy; Myofascial Release |
| Traction | Mechanically lengthening muscle and fascia by pulling on joints. | Everywhere. | Craniosacral; Sports Massage; Structural Integration |
| **V**- Flopping | Limb is lifted and bounced (with support for joints) on the table. | Arms and legs. | Sports Massage; Swedish; Trager |
| **V** - Rocking or jostling | Slower rhythmic type of vibration applied to the whole body in conjunction with the patient’s body rhythm. | Everywhere. | Swedish; Trager; Tuina; Shiatsu |
| **V** – Shaking | A type of vibration, using a lifting or pulling of the skin or a limb and then rhythmically shaking it. | Everywhere, except the face. | Swedish; Trager |
| **V** – Vibration | Continuous shaking or trembling movement made by hands, fingers or mechanical tool that is focused on a specific area of the body. Vibration can be applied to the entire back by moving the hands, fingers, or tool systematically across it. | Everywhere. | Swedish;  Tuina |

*Techniques are listed alphabetically, except that those considered “closely related” are grouped together and can be distinguished because they have one or more capitalized letters in front of them. The letter is NOT part of the technique’s name.

** Although common parlance among massage therapists when describing specific techniques [23, 24], the terms “Post-Isometric Relaxation” and “Reciprocal Inhibition” technically refer to the originally purported mechanism of those techniques [25]. The work of Ballentyne et al [25] has shown that the purported mechanisms do not accurately explain the observed effects.
